# Food waste measurement in Australian hospitals and residential aged care homes

**DOI:** 10.3389/fnut.2025.1715385

**Published:** 2025-12-05

**Authors:** Nathan Cook, Kate Sansome, Karly Bartrim, Jorja Collins

**Affiliations:** 1 School of Human Movement and Nutrition Sciences, Faculty of Health, Medicine and Behavioural Sciences, The University of Queensland, Brisbane, QLD, Australia; 2 Adelaide Business School, Faculty of Arts, Business, Law and Economics, The University of Adelaide, Adelaide, SA, Australia; 3 Department of Nutrition, Dietetics and Food, Faculty of Medicine, Nursing and Health Sciences, Monash University, Melbourne, VIC, Australia

**Keywords:** food waste, hospital, aged care, foodservice, sustainability, Australia

## Abstract

Food waste in Australian hospitals and Residential Aged Care Homes (RACH) poses a significant challenge to building a national sustainable food system. These settings generate substantial amounts of food waste due to the complex interaction between patient and resident needs, foodservice operations, staff behaviour and attitudes, food safety regulations and nutrition policies. Measuring food waste is essential to establish baseline waste metrics, guide targeted reduction strategies and evaluate the success of waste reduction efforts in meeting Australia’s national strategy to halve food waste by 2030. Integrating food waste measurement in hospitals and RACH can reduce waste and provide positive environmental and financial outcomes. However, measurement practices in these settings currently use labour-intensive methods, often lacking digital integration, and are conducted ad-hoc as quality improvement activities. Overarching hospital and RACH policy in Australia governing the quality of food and nutrition care is improving and may support further enhancements in waste measurement, waste management and waste reduction. Nonetheless, individual hospitals and RACH foodservices remain responsible for driving change. There is an urgent need to improve food waste measuring practices in these Australian settings to achieve significant reductions in food waste.

## 1 Introduction

Foodservices contribute over a quarter of the food waste at the consumption end of the food supply chain (1). Hospital and Residential Aged Care Home (RACH) foodservices have the complex task to provide their unique and diverse patient and resident populations safe, nutritious and enjoyable food at scale and within budget restrictions (2). Hospital and RACH foodservices produce large quantities of food waste (3). Food waste occurs at the various points of the foodservice system, including food preparation, cooking, plating, and service/consumption (2, 4). Food waste has been reported as 50% of total hospital waste (5–7), and in RACHs, 23–50% of food prepared is wasted (8). Contributing factors to food waste are divided into patient/resident aspects, foodservice system design limitations, staff attitudes, food safety regulations and nutritional provision policies (9). In hospitals, patients may have low appetite and minimal interest in food from their clinical condition (10), whereas in RACHs reduced function or cognition from ageing impacts food consumption (11). Foodservice system characteristics contributing to food waste which impact both hospitals and RACHs include limited food choice, poor food quality and appearance, limited variety, inappropriate meal times, and delays between ordering and receiving food (10, 12–17). Across both these settings, overall patient/resident satisfaction with the foodservice is consistently related to food quality (18). Hospital and RACH staff have also been reported to feel undervalued, undertrained, risk adverse and sceptical to change, decreasing their motivation to participate in food waste reduction initiatives (9, 19, 20). Regulation and policy governing these settings prioritises patient and resident safety and clinical outcomes, which can lead to unintended food waste. For example, hospitals repeatedly aim to provide patients with sufficient food to meet their requirements to prevent malnutrition, which is often more food than patients are able to consume (9). With multiple causes for food waste in these settings food waste reduction in hospitals and RACHs is imperative.

Measuring food waste can provide insight into baseline levels of waste and problem areas to support the design of targeted waste reduction efforts implemented through quality improvement activities (21). Measuring waste might explore the total amount of waste, differences in food prepared and served from overproduction and incorrect forecasting, plate waste data (i.e., uneaten food served to patients/residents), and (un)popular menu items (22). Data collectors observe and or collect, separate, and measure (weigh or count) food waste from particular points in the foodservice system at planned data collection times (i.e., during/after service) to generate the information of interest (22). The data on the amount of food waste (e.g., weight or number of items) is valuable in and of itself, but it can also be converted into other meaningful outcomes. Environmental indicators such as carbon emissions or water footprint can be estimated via tools such as the ReFED impact calculator (23, 24). Financial outcomes can also be derived through cost calculations (i.e., cost of wasted food, disposal costs) (25). Data from individual audits can then be extrapolated to identify significant opportunities for service improvements that result in environmental and financial savings. For example, one Australian hospital foodservice was spending $22,816.15/year sending food and packaging waste to landfill (25) and a Candadian RACH estimated 40% of their annual food budget going to waste (26). Clearly, unrealised efficiencies can be identified from measuring food waste to promote sustainable outcomes.

In this perspective, we discuss why food waste measurement is important and how food waste reduction can occur in hospitals and RACHs. We hope to promote regular food waste measurement in Australian hospitals and RACH foodservices that leads to subsequent efforts in reduction.

## 2 Subsections

### 2.1 Why hospitals and RACH should measure food waste

The environmental, economic and social impacts of food waste have been well documented globally (27). When food is wasted in landfills, greenhouse gases are produced, there is a loss of natural resources along the food supply chain, businesses and consumers incur financial losses, and food insecurity, undernourishment, hunger and injustice are exacerbated (27). These outcomes emphasise the importance of The United Nations Sustainable Development Goal 12.3 (SDG 12.3), to *“By 2030, halve per capita global food waste….”* (28, 29). Unfortunately, progress towards the goal is failing and with less than 5 years until 2030, time is running out (30). Countries, including Australia, have committed to the cause and endeavour to reach the target (31).

Australia’s 2017 National Food Waste Strategy provided a framework to support collective action to halve Australia’s food waste in line with SDG 12.3’s 50% reduction target (31). As part of the strategy, Food Innovation Australia were commissioned to develop a roadmap and feasibility study to achieve this target (32). Measuring food waste was identified as the second most impactful industry-led intervention recommended to reduce food waste measured in total tonnes of food waste reduced, behind the development and voluntary participation in sector action plans. This highlights the important role of measuring food waste in establishing baseline waste levels and informing reduction strategies.

### 2.2 How to measure food waste

Measuring food waste through a ‘food waste audit’, in hospitals and RACHs is typically completed cross-sectionally. Data collectors are often foodservice staff members or tertiary health profession students undertaking placement. Traditionally, plate waste audits have been commonplace, focusing on evaluating patient or resident meal satisfaction and nutrition intake. Two common manual methods are used: (i) visual estimation via observing plate waste and assigning an amount consumed (e.g., 0, 25, 50, 75, 100%), or (ii) weighing plate waste via scales for greater accuracy (22). For example, a 3 day plate waste audit using visual estimation in a 33 bed Canadian RACH estimated 28% waste on average (33); and a 5 day plate waste audit using scales in a 250 bed American hospital captured 1,098 kgs (34). In recent years, waste audits that look beyond plate waste have increased. In Australia, audits have been conducted in hospitals that measure total waste (25), packaging waste (35, 36) or production waste (36), using floor scales and large bins to weigh food waste. In comparison, completion of food waste audits in RACHs are less common (37).

Waste audits are usually labour-intensive due to the large amount of waste generated and the time, physical exertion and coordination required to manually collect, sort, measure and dispose of the waste, accurately and as fast as possible (22). Conflicting time-sensitive operational responsibilities of staff rarely allows waste measurement to be prioritised, as the foodservice is continuously preparing for the next meal or cleaning up from the last (20). Detailed planning is required to understand what the tasks are, the team involved, audit duration, collection strategy, measurement method, equipment required, and outcomes of interest. To support hospital staff in audit design, a consensus pathway food waste audit tool has been developed to recognise the decisions needed to be made depending on the type of audit being planned (22). The focus of the tool is to consider the variables listed above to customise an audit that allows food waste measurement to be targeted to the site’s needs, and repeated over time. This tool is also translatable to RACHs.

The burdens of measuring food waste are likely to decrease with the introduction of new technologies. Advancements in Artificial Intelligence (AI) have led to the development of commercial food waste measurement technology which may simplify, speed up and encourage waste measurement (38). Currently available products include a combination of hardware/software solutions that photograph, screen and weigh food waste in seconds, with data stored in cloud-based progress dashboards. There has been some local success in hospitals (39) and RACHs in Australia (40) and further internationally (41), as well as in other settings, including hospitality and tourism (42). However, these technologies appear to not be widely adopted in Australian hospitals and RACHs. Leanpath is an example of an AI camera bin technology which is complementary to Australia’s most popular hospital foodservice menu management software CBORD (43, 44). With the increasing prevalence and acceptance of AI tools in workplaces, there is an opportunity for these tools to improve food waste data collection capacity and accuracy. New technologies including AI measurement tools are likely to solve common barriers to food waste audits, such as staff buy-in, time, labour costs, reliance on students, and detailed planning, while simultaneously training foodservice staff in new skills (19, 20).

End Food Waste Australia (EFWA) (45) is Australia’s leading organisation for improving food waste and has just released its Institutions Sector Action Plan for Hospital and Aged Care (9). The researchers completed a literature review, interviews, site visits and co-design workshops with hospital and RACH stakeholders to identify food waste hot spots, root causes of food waste and find targeted solutions to prioritise for implementation. The findings highlighted the critical role of measurement and ongoing monitoring and evaluation to reduce food waste. Sites that embedded systematic waste measurement into routine practice were better positioned to implement targeted interventions that enhanced patients’ food service experiences and reduced waste (46). Recognising that all hospitals and RACHs have different circumstances and resources available, their recommended approach for sites to improve measurement practices is to start within their current capacity, as small realistic changes can have large impacts over time. Sites should begin with basic awareness and engagement (i.e., visual bin inspections), move towards intermediate tracking and evaluation (i.e., weighing waste using pre-marked containers) and finally upgrade to using advanced data-driven monitoring (i.e., AI technology) (9).

### 2.3 How to reduce food waste

There are a range of strategies that can be implemented to reduce food waste, depending on the type of waste and why it occurs. Many reviews have been published describing these approaches and the evidence of their success (3, 16, 17, 47, 48). Examples of these reduction strategies in hospitals include: flexible portion sizes (15), increased food choice (15), altering foodservice system models (17), better food presentation (17), and higher food quality (16). For example, two hospitals were able to reduce food waste by 13% (49) and 17% (50) respectively after moving to a room-service model (i.e., order on demand). Additionally, the use of electronic bed side meal ordering systems delivered verbally by staff to patients compared to paper based menu ordering completed by patients alone reduced waste by 4% (51). In RACHs, there is less comprehensive research with a recent scoping review finding only 3 examples of food waste reduction strategies from their 33 sources (37). Two of these studies found pre-post food waste reduction outcomes from; redesigning dinnerware (52), which reduced waste from 4 to 17% depending on the plate colour; and using staff co-design focus groups targeting resource efficiency, which reduced waste by 8% (53). Further, a food production kitchen that produces food for 14 RACH sites used a government-designed ‘love food hate waste’ toolkit to measure waste over 6 weeks, introducing several interventions (e.g., portion sizes) that reduced their baseline food waste sent to landfill by half (54). The EFWA Sector Action Plan (9) also identified reduction strategies including engaging directly with patients/residents to understand reasons for uneaten food, digital procurement systems to calculate exact ingredients required for recipes, upcycling food waste into new recipes (i.e., bananas to banana bread), updating RACH resident meal preferences more often and embedding food waste training in hospital staff onboarding processes. Waste measurement data can also be used to drive menu item and portion size refinements which can be facilitated using co-design between foodservice, clinical and care staff.

### 2.4 Policy influencing food waste measurement in hospitals and RACHs

Currently, the overarching Australian National Safety and Quality Health Service Standards do not address environmental sustainability (55). A recent comprehensive review of sustainable healthcare policy in Australia identified a wide range of policies being implemented by individual healthcare service providers in different states/territories (56). These examples came from members of the Australian Global Green and Healthy Hospitals network. Some environmental policies were focused on specific sustainability priorities, including the Ambulance Victoria Climate Strategy and Tasmanian Energy Audit of Health Agencies (56). Most policies found were state-wide healthcare net-zero emission reduction targets or climate/sustainability roadmaps, with no specific policies on food alone. Only 23% of the 439 hospitals and 1687 health service providers surveyed reported working on a sustainable food concept. Yet, food was only mentioned in two of the named policies, describing local food production and supply of healthy food in public healthcare settings. Many of the policies had not yet been adopted/implemented within healthcare organisations. Separately, the additional academic literature review from this work found only four publications of the total 37 included reporting sustainable food strategies. Examples focusing on food waste reduction were the redistribution of unused food to charity, the reuse of unopened oral nutrition supplements, the implementation of room service, and reducing purchasing volumes. Despite these positive examples in practice, there is opportunity to make explicit food waste policy commitments in Australian hospitals and RACHs. This research indicates that the current Australia-wide sustainable healthcare module being developed by the Australian Commission on Safety and Quality in Health Care should consider sustainable food practices, including food waste measurement.

At the state policy level, three out of eight Australian state/territories policies for food and nutrition in hospitals and RACHs include recommendations regarding food waste. The Queensland Foodservice Best Practice Guideline reports text describing the frequency of measuring food waste twice per year (57). The Victorian Nutrition and Food Quality Standards for Adults in Public Hospitals and Residential Aged Care Services recommend food waste measurement once per year (58). The New South Wales Nutrition Standards acknowledge the importance of reducing food waste; but do not mention measurement frequency (59). These documents previously only considered nutrition care and now incorporate recommendations for improvements in service quality and sustainability. This allows hospitals in these states to prioritise food waste measurement.

Similar recommendations have not been widely replicated specifically for RACH. The Strengthened Australian Aged Care Quality Standards are coming into effect in 2025 following the recommendation from the 2021 Royal Commission into Aged Care Quality and Safety (60, 61). For the first time, these standards include a whole standard on Food and Nutrition (Standard Six) (62). This standard focuses on the resident’s perspective, and if they believe they are receiving plenty of food and drinks they enjoy; that food and drinks are nutritious, appetising, safe, and meet their needs and preferences; and that the dining experience is enjoyable, includes variety and supports a sense of belonging. In the future, it is hoped that RACHs will follow the progress made in hospitals regarding food waste, particularly given residents have identified concerns for food waste (63).

The Sector Action Plan from EFWA may play a significant role driving practice change in Australian hospitals and RACHs (9). Their recommendations were targeted towards government, policy makers, and individual hospitals and RACHs. From a 5% reduction in food waste based on the report’s actions, Australian hospitals and RACHs can prevent 10,703 tonnes of food waste by 2048. The Sector Action Plans release hopefully demonstrates to the sector the value of regular measurement in practice.

## 3 Discussion

Australia has a national target of 50% food waste reduction by 2030 (29). Measuring food waste is an effective starting point to reduce food waste (9, 22). Hospitals and RACHs should therefore be encouraged to measure food waste and realise their responsibility to contribute to the sector’s capacity for large systemic food waste reduction. This perspective piece has discussed current food waste measurement practices in hospitals and RACHs, highlighted possible solutions that may reduce food waste and commented on recent changes in policy in Australian hospitals and RACHs. While individual hospital and RACH foodservices appear responsible for driving change, using guided direction from the Sector Action Plan (9) with internal organisation executives, industry solutions and government bodies is warranted (64). To make significant progress, regular planned and scheduled food waste measurement activities using customised methods can guide enhanced action to reduce food waste through necessary quantification of waste amounts and subsequent efforts to reduce it.

### 3.1 Behaviour change research

It is recognised that there is a complexity to measuring food waste in hospital and RACH foodservices, impacting efficient operations (19, 20, 48). In hospitals, limited financial incentive, low resource availability, staff attitudes and time constraints limit the capacity of already prioritised daily tasks to be replaced for food waste measurement activities (19, 20). Whereas in RACHs food waste measurement is not a current priority, due to extensive pressure and reform on the sector, such as staff shortages, more reporting requirements and changing legislation and standards for resident food choice/nutritional requirements (37, 65). To counteract these competing sector demands future research could design, test, and evaluate behaviour change interventions that activate the right leverage points in these settings to promote food waste measurement. These interventions will need to be targeted at each level of the sector: patient/resident (micro), organisation (meso) and policy (macro) and consider a co-design approach (9). Previous qualitative research with hospital foodservice staff referred to the Theoretical Domains Framework, Capability-Opportuninty-Motivation-Behaviour model, and the Behaviour Change Wheel identifying education, training, environmental restructuring, enablement and modelling as the strongest intervention types to enable change (19). Leaning on pre-established behaviour change frameworks such as the Behaviour Change Taxonomy (66) which aligns with these previous frameworks used would be essential to strengthen the exploration and could highlight more effective strategies favourable to the healthcare context. This may empower foodservices teams to prioritise food waste measurement and sector wide sharing of the outcomes via industry connections could accelerate collective progress.

### 3.2 Collaborative learning

Strategic partnerships can support hospital and RACH organisations in piloting strategies that may improve their food waste measurement and reduction activities. In Australia, the Climate and Health Alliance leads the Global Green and Healthy Hospitals (GGHH) initiative, of which food is one of its action areas (67). The GGHH in Australia delivers a community of practice that provides information sharing, practical tips, and space for discussion with industry, academics, and health sector representatives. Their online trove of case studies is another resource for learning and sharing. However, there are gaps in opportunities for RACH organisations to meet and share. Other organisations, including the Lantern Alliance (RACH industry body) (68), Leanpath (41), Institute of Hospitality and Healthcare (healthcare staff industry body) (69) and EFWA (45) have previously delivered online workshops and in-person conferences to train the RACH workforce and share expertise. Hospital and RACH leaders should encourage their organisations/staff to be involved in these opportunities and bridge the science to practice implementation gap, further strengthening progress towards food waste measurement and reduction.

## 4 Conclusion

There is an urgent need to change food waste measurement practices in Australian hospitals and RACHs. Despite clear benefits, measuring food waste through traditional food waste audits is difficult to execute due to the time requirements and reliance on manual labour, though technology may reduce this barrier. Hospital and RACH foodservices are strongly encouraged to embed food waste measurement activities into regular practice that provide them with a baseline understanding of their food waste levels, that will help them develop further plans for reduction. Establishing evidence of successful food waste measurement and subsequent reduction moving forward as well as sharing these outcomes with hospital and RACH foodservices, organisation executives, and policy makers, may create a shift in the status quo. This process may be enhanced through the application of well-designed behaviour change campaigns. Sector-wide implementation of these activities could substantially reduce the sector’s climate footprint and help achieve Australia’s national goal of halving food waste by 2030 in line with SDG 12.3 (28, 29).

## Data Availability

The original contributions presented in the study are included in the article/supplementary material, further inquiries can be directed to the corresponding authors.

## References

[1] United Nations Environment Programme (UNEP). Food waste index report 2021 Nairobi: UNEP; (2021). Available online at: https://www.unep.org/resources/report/unep-food-waste-index-report-2021 (Accessed June 24, 2025).

[2] do Rosario VA Walton K. Hospital food service In: H. L. Meiselman (ed.). Handbook of eating and drinking. Switzerland: Springer (2019). 1–27.

[3] Carino S Porter J Malekpour S Collins J. Environmental sustainability of hospital foodservices across the food supply chain: a systematic review. J Acad Nutr Diet. (2020) 120:825–73. doi: 10.1016/j.jand.2020.01.001, PMID: 32093919

[4] Cook N Goodwin D Porter J Collins J. Food and food-related waste management strategies in hospital food services: a systematic review. Nutr Diet. (2023) 80:116–42. doi: 10.1111/1747-0080.12768, PMID: 36168297

[5] Anari R Nikooyeh B Ghodsi D Amini M Neyestani TR. An in-depth analysis of hospital food waste in terms of magnitude, nutritional value, and environmental and financial perspectives: a cross-sectional study. Waste Manag Res. (2024) 42:167–77. doi: 10.1177/0734242X231176733, PMID: 37300389

[6] Alam MM Sujauddin M Iqbal GMA Huda SMS. Report: healthcare waste characterization in Chittagong medical college hospital. Bangladesh Waste Manag Res. (2008) 26:291–6. doi: 10.1177/0734242X07087661 18649578

[7] Mattoso VD Schalch V. Hospital waste management in Brazil: a case study. Waste Manag Res. (2001) 19:567–72. doi: 10.1177/0734242X0101900613 12201687

[8] McAdams B Robinson E Gordon R. Investigating food waste generation at long-term care facilities in Ontario. BFJ. (2023) 125:2902–17. doi: 10.1108/BFJ-06-2022-0561

[9] Sansome K. Willmott T. Baker J. Conduit J. (2025). Hospital and aged care sector action plan 2025. End food waste Australia. Available online at: https://endfoodwaste.com.au/wp-content/uploads/2025/07/Hospitals-Aged-Care-Report-Summary_Web.pdf (Accessed on August 1, 2025)

[10] Porter J Collins J. A qualitative study exploring hospital food waste from the patient perspective. J Nutr Educ Behav. (2021) 53:410–7. doi: 10.1016/j.jneb.2020.10.008 33526388

[11] Gaskill D Black LJ Isenring EA Hassall S Sanders F Bauer JD. Malnutrition prevalence and nutrition issues in residential aged care facilities. Australas J Ageing. (2008) 27:189–94. doi: 10.1111/j.1741-6612.2008.00324.x, PMID: 19032620

[12] Bernaert N De Rycke E Hunninck M Vlaemynck G Van Pamel E Vangeyte J . Monitoring plate and preparation food waste in residential facilities for elderly people: a case study in Flanders (Belgium). Waste Manag. (2025) 195:189–99. doi: 10.1016/j.wasman.2025.02.013 39923656

[13] Zilujko J Abbey K Capra S. Exploring and understanding perceptions and definitions of foodservice quality in residential aged care: a scoping review. Nutr Diet. (2025). doi: 10.1111/1747-0080.70005, PMID: 40190257 PMC12884207

[14] Stephens LD Porter J Lawrence M. Healthy and environmentally sustainable food procurement and foodservice in Australian aged care and healthcare services: a scoping review of current research and training. Sustain. (2021) 13:11207. doi: 10.3390/su132011207

[15] Antasouras G Vasios GK Kontogiorgis C Ioannou Z Poulios E Deligiannidou GE . How to improve food waste management in hospitals through focussing on the four most common measures for reducing plate waste. Int J Health Plann Manag. (2023) 38:296–316. doi: 10.1002/hpm.3586, PMID: 36193027

[16] Mahmoudifar K Raeesi A Kiani B Rezaie M. Food waste in hospitals: implications and strategies for reduction: a systematic review. MEQ. (2025) 36:50–71. doi: 10.1108/MEQ-07-2023-0221

[17] Rinninella E Raoul P Maccauro V Cintoni M Cambieri A Fiore A . Hospital services to improve nutritional intake and reduce food waste: a systematic review. Nutrients. (2023) 15:310. doi: 10.3390/nu15020310, PMID: 36678180 PMC9864175

[18] Lai H Gemming L. Approaches to patient satisfaction measurement of the healthcare food services: a systematic review. Clin Nutr ESPEN. (2021) 1:61–72. doi: 10.1016/j.clnesp.2020.12.019 33745623

[19] Cook N Collins J Porter J Goodwin D. Applying the theoretical domains framework and behavior change wheel to inform interventions for food and food-related waste audits in hospital foodservices. Front Nutr. (2023) 10:1204980. doi: 10.3389/fnut.2023.1204980, PMID: 37654474 PMC10465701

[20] Cook N Collins J Goodwin D Porter J. Factors influencing implementation of food and food-related waste audits in hospital foodservices. Front Nutr. (2022) 9:1062619. doi: 10.3389/fnut.2022.1062619, PMID: 36532534 PMC9753938

[21] Amicarelli V Bux C. Food waste measurement toward a fair, healthy and environmental-friendly food system: a critical review. BFJ. (2020) 123:2907–35. doi: 10.1108/BFJ-07-2020-0658

[22] Cook N Collins J Goodwin D Porter J. A systematic review of food waste audit methods in hospital foodservices: development of a consensus pathway food waste audit tool. J Hum Nutr Diet. (2022) 35:68–80. doi: 10.1111/jhn.12928, PMID: 34060673

[23] ReFED. Impact calculator. (2025). Available online at: https://insights-engine.refed.org/impact-calculator (Accessed August 1 2025)

[24] Yip YMJ Cook N Collins J. Food waste management practices in hospital foodservices and their associated greenhouse gas emissions: potential for increased environmental sustainability. Front Nutr. (2025) 12:1541657. doi: 10.3389/fnut.2025.1541657, PMID: 40432959 PMC12106006

[25] Collins J Porter J. Quantifying waste and its costs in hospital foodservices. Nutr Diet. (2023) 80:192–200. doi: 10.1111/1747-0080.12796, PMID: 36690908

[26] Liu Y Oulton J. A food waste monitoring project in a long term care facility in Ontario. Can J Diet Pract Res. (2016) 77:e10–1.

[27] Seberini A. Economic, social and environmental world impacts of food waste on society and zero waste as a global approach to their elimination. SHS Web of Conferences. (2020) 74:10. doi: 10.1051/shsconf/20207403010

[28] United Nations - Department of Economic and Social Affairs. Goal 12: ensure sustainable consumption and production patterns – targets and indicators. (2025). Available online at: https://sdgs.un.org/goals/goal12#targets_and_indicators (Accessed June 24 2025)

[29] Lipinski B. SDG target 12.3 on food loss and waste: 2024 Progress report. (2024). Available online at: https://champions123.org/sites/default/files/2024-09/champions-12-3-2024-progress-report.pdf (Accessed June 24 2025)

[30] United Nations Department of Economic and Social Affairs. The sustainable development goals report 2025. (2025). Available online at: https://unstats.un.org/sdgs/report/2025/ (Accessed September 5 2025)

[31] Commonwealth of Australia. (2017). National Food Waste Strategy: halving Australia’s food waste by 2030. Available online at: https://www.dcceew.gov.au/sites/default/files/documents/national-food-waste-strategy.pdf (Accessed June 24 2025)

[32] Food Innovation Australia (FIAL). The National Food Waste Strategy Feasibility Study - final report. (2021). Available online at: https://www.fial.com.au/sharing-knowledge/food-waste (Accessed June 21, 2021)

[33] Miller C Taylor J Tong R Thompson S Thomson E Robertson A . Something to chew on; plate-waste at an Ontario veteran’s Centre. Can J Diet Pract Res. (2024) 85:106–10. doi: 10.3148/cjdpr-2024-007, PMID: 38832646

[34] Freedman MR Franklin IB. Implementing a solid waste management diversion program in a conventional cook–serve hospital system: a feasibility study. J Hunger Environ Nutr. (2010) 5:370–9. doi: 10.1080/19320248.2010.504109

[35] Hall L Dow A Burns K Sadek J Sikovska D Allen A . The environmental impact of tray-line packaging waste at an Australian tertiary hospital: retrospective observational study. Nutr Diet. (2025) 35:370–379. doi: 10.1111/1747-0080.70028, PMID: 40640086 PMC13575692

[36] Cook N Habel J McCray S Utter J Brennan K. Quantifying and describing production waste in two urban healthcare centres with differing foodservice models. Nutr Diet. (2025) 82:434–44. doi: 10.1111/1747-0080.70013, PMID: 40176755 PMC12401807

[37] Roulston M Thompson C Pelly F Cave D. Food waste in residential aged care: a scoping review. Nutr Diet. (2025). doi: 10.1111/1747-0080.70034 PMC12884254 40878785

[38] Bux C. Conventional and digital technologies for measuring and monitoring food waste in the healthcare foodservice. J Foodserv Bus Res. (2024) 27:1–23. doi: 10.1080/15378020.2024.2394730

[39] Smith S. Compass group sustainability report highlights Australia's 34% food waste reduction with Leanpath. (2025). Available online at: https://blog.leanpath.com/compass-group-sustainability-report-highlights-australias-34-food-waste-reduction-with-leanpath (Accessed June 26 2025)

[40] AerVision Technologies. AerMeal® V2 – AI- & Machine Learning-Enabled Food Intake Monitoring. Siemens / AerVision technologies. (2025). Available online at: https://assets.new.siemens.com/siemens/assets/api/uuid:0d6da219-267a-4ff7-b402-a6980250697e/aermeal-v2.pdf (Accessed September 1 2025)

[41] Leanpath. (2025). Hospital food waste management solutions. Available online at: https://www.leanpath.com/industries/hospital-food-waste-management-solutions/ (Accessed September 1 2025)

[42] Leanpath. Industries. (2025). Available online at: https://www.leanpath.com/industries/ (Accessed June 23 2025)

[43] Reichard S. Preventing food waste with Leanpath and CBORD integration. (2023). Available online at: https://www.cbord.com/all-wb-preventing-food-waste-with-leanpath-and-cbord-integration/?source=blog-healthcare (Accessed June 25 2025)

[44] Leanpath. (2023). Leanpath integrates with CBORD to deliver more precise and actionable food waste data. Available online at: https://blog.leanpath.com/leanpath-integrates-with-cbord-to-deliver-more-precise-and-actionable-food-waste-data (Accessed June 26 2025)

[45] End Food Waste Australia (EFWA). End food waste Australia. (2025). Available online at: https://endfoodwaste.com.au/ (Accessed August 23 2025)

[46] Sansome K Willmott T Baker J Conduit J. A sector-wide investigation into food waste in hospitals and aged care In: Proceedings of the 41st national IHHC conference. Perth: Institute of Hospitality In Healthcare (2024)

[47] Alshqaqeeq F Twomey JM Overcash MR. Food waste in hospitals. IJHTM. (2018) 17:186–96. doi: 10.1504/IJHTM.2018.098389

[48] Torrejón-Ramos M Medina-Salgado M-S Ortiz-de-Urbina-Criado M. A scoping review on reducing food waste and loss in hospitals. Gac Sanit. (2025) 39:102462. doi: 10.1016/j.gaceta.2025.102462, PMID: 39985827

[49] McCray S Maunder K Barsha L Mackenzie-Shalders K. Room service in a public hospital improves nutritional intake and increases patient satisfaction while decreasing food waste and cost. J Hum Nutr Diet. (2018) 31:734–41. doi: 10.1111/jhn.12580, PMID: 29989236

[50] McCray S Maunder K Krikowa R. Room service improves nutritional intake and increases patient satisfaction while decreasing food waste and cost. JAND. (2018) 118:284–93. doi: 10.1016/j.jand.2017.05.014 28676228

[51] McCray S Maunder K Norris R Moir J MacKenzie-Shalders K. Bedside menu ordering system increases energy and protein intake while decreasing plate waste and food costs in hospital patients. Clin Nutr ESPEN. (2018) 26:66–71. doi: 10.1016/j.clnesp.2018.04.012, PMID: 29908685

[52] Hansen KV Derdowski LA. Sustainable food consumption in nursing homes: less food waste with the right plate color? Sustain. (2020) 12:6525. doi: 10.3390/su12166525

[53] Strotmann C Friedrich S Kreyenschmidt J Teitscheid P Ritter G. Comparing food provided and wasted before and after implementing measures against food waste in three healthcare food service facilities. Sustain. (2017) 9:1409. doi: 10.3390/su9081409

[54] Love Food Hate Waste. Aged care program. (2024). Available online at: https://www.lovefoodhatewaste.nsw.gov.au/agedcare (Accessed August 10 2025)

[55] Australian Commission on Safety and Quality in Health Care. National Safety and quality health service (NSQHS) standards 2nd ed. (2021). Available online at: https://www.safetyandquality.gov.au/sites/default/files/2021-05/national_safety_and_quality_health_service_nsqhs_standards_second_edition_-_updated_may_2021.pdf (Accessed September 5 2025)

[56] Wyns A BP Armstrong F Carino S Dolker D Lennox A Tsering D. A review of sustainable healthcare: policy, practice, and research with a focus on safety and quality. (2022). Available online at: https://www.safetyandquality.gov.au/sites/default/files/2022-10/a_review_of_sustainable_healthcare_-_june_2022.pdf (Accessed September 4 2025)

[57] Queensland Health. Foodservice best practice guideline. (2023). Available online at: https://www.health.qld.gov.au/__data/assets/pdf_file/0023/655340/qh-gdl-448.pdf (Accessed August 20 2025)

[58] Victorian Department of Health. (2021). Nutrition and food quality standards for health services. 2021. Available online at: https://www.health.vic.gov.au/quality-safety-service/nutrition-and-food-quality-standards-for-health-services (Accessed August 21 2025)

[59] Agency for Clinical Innovation. Sustainability. (2024). Available online at: https://aci.health.nsw.gov.au/projects/nutrition-standards/about/sustainability (Accessed August 29 2025)

[60] Aged Care Quality and Safety Commission. Quality standards. (2024). Available online at: https://www.agedcarequality.gov.au/providers/quality-standards (Accessed August 19 2025)

[61] Australian Government Department of Health Disability and Ageing. (2025). About improving food and nutrition in aged care. Available online at: https://www.health.gov.au/our-work/improving-food-nutrition-aged-care/about (Accessed August 25 2025)

[62] Australian Government Aged Care Quality and Safety Commission. Standard 6: food and nutrition. (2025). Available online at: https://www.agedcarequality.gov.au/strengthened-quality-standards/food-and-nutrition (Accessed September 29)

[63] Australian Government Aged Care Quality and Safety Commission. Analysis of a survey of food and dining experiences in residential aged care services - final report. (2023). Available online at: https://www.agedcarequality.gov.au/resource-library/analysis-survey-food-and-dining-experiences-residential-aged-care-services-final-report (Accessed 29 September 2025)

[64] Organisation for Economic Co-operation and Development (OECD). A stocktaking of food loss and waste policies: Australia. (2025). Available online at: https://www.oecd.org/content/dam/oecd/en/topics/policy-issue-focus/food-loss-and-waste/A%20Stocktaking%20of%20FLW-Australia.pdf (Accessed June 24 2025)

[65] Cave D Abbey K Capra S. The challenges facing residential aged care homes to participate in quality food and nutrition research. J Hum Nutr Diet. (2023) 36:1547–55. doi: 10.1111/jhn.13154, PMID: 36752077

[66] Michie S Richardson M Johnston M Abraham C Francis J Hardeman W . The behavior change technique taxonomy (v1) of 93 hierarchically clustered techniques: building an international consensus for the reporting of behavior change interventions. Ann Behav Med. (2013) 46:81–95. doi: 10.1007/s12160-013-9486-6, PMID: 23512568

[67] Climate and Health Alliance. Climate and Health Alliance. (2025). Available online at: https://www.caha.org.au/ (Accessed September 1 2025)

[68] Lantern Alliance. (2025). Lantern Alliance. Available online at: https://lanternalliance.com.au/ (Accessed September 5 2025)

[69] Institute of Hospitality in HealthCare. Institute of Hospitality in HealthCare Ltd. (2025). Available online at: https://www.ihhc.org.au/ (Accessed Septermber 5 2025)

